# CdS Nanoparticle-Modified α-Fe_2_O_3_/TiO_2_ Nanorod Array Photoanode for Efficient Photoelectrochemical Water Oxidation

**DOI:** 10.1186/s11671-017-2278-3

**Published:** 2017-09-02

**Authors:** Ruiyang Yin, Mingyang Liu, Rui Tang, Longwei Yin

**Affiliations:** 1 School of Physics, Shandong University, Jinan, 250100 People’s Republic of China; 2 Key Laboratory for Liquid–Solid Structural Evolution and Processing of Materials, Ministry of Education, School of Materials Science and Engineering, Shandong University, Jinan, 250061 People’s Republic of China

**Keywords:** TiO_2_, α-Fe_2_O_3_, CdS, Nanorod, Photoelectrochemical Water Oxidation

## Abstract

In this work, we demonstrate a facile successive ionic layer adsorption and reaction process accompanied by hydrothermal method to synthesize CdS nanoparticle-modified α-Fe_2_O_3_/TiO_2_ nanorod array for efficient photoelectrochemical (PEC) water oxidation. By integrating CdS/α-Fe_2_O_3_/TiO_2_ ternary system, light absorption ability of the photoanode can be effectively improved with an obviously broadened optical-response to visible light region, greatly facilitates the separation of photogenerated carriers, giving rise to the enhancement of PEC water oxidation performance. Importantly, for the designed abnormal type-II heterostructure between Fe_2_O_3_/TiO_2_, the conduction band position of Fe_2_O_3_ is higher than that of TiO_2_, the photogenerated electrons from Fe_2_O_3_ will rapidly recombine with the photogenerated holes from TiO_2_, thus leads to an efficient separation of photogenerated electrons from Fe_2_O_3_/holes from TiO_2_ at the Fe_2_O_3_/TiO_2_ interface, greatly improving the separation efficiency of photogenerated holes within Fe_2_O_3_ and enhances the photogenerated electron injection efficiency in TiO_2_. Working as the photoanodes of PEC water oxidation, CdS/α-Fe_2_O_3_/TiO_2_ heterostucture electrode exhibits improved photocurrent density of 0.62 mA cm^− 2^ at 1.23 V vs. reversible hydrogen electrode (RHE) in alkaline electrolyte, with an obviously negatively shifted onset potential of 80 mV. This work provides promising methods to enhance the PEC water oxidation performance of the TiO_2_-based heterostructure photoanodes.

**Electronic supplementary material:**

The online version of this article (10.1186/s11671-017-2278-3) contains supplementary material, which is available to authorized users.

## Background

To solve the severe problem of pollution and limited resources of fossil, photoelectrochemical (PEC) water splitting to produce hydrogen has been regarded as one of the most promising strategies for solar energy conversion. Since the first report on PEC water oxidation based on TiO_2_ [1], TiO_2_ has drawn much attention as the photoanode materials for PEC water oxidation, due to its stable PEC properties, strong optical response, and suitable energy band position [2, 3]. However, the PEC performance of pristine TiO_2_ photoanode is greatly confined by the slow water oxidation kinetics originated from the poor photogenerated carrier separation capability and insufficient light absorption ability [4, 5].

Therefore, various strategies have been taken to improve the PEC water oxidation performance of pristine TiO_2,_ such as surface modification [6], quantum dot sensitization, and heterojunction construction [7, 8]. One efficient method to improve the photogenerated carrier separation performance is to construct heterostructured photoanode. For instance, constructing heterojunction between TiO_2_ and other metal oxide semiconductors with matched energy band structures (like Co_3_O_4_/TiO_2_ [9] and ZnIn_2_S_4_/TiO_2_ [10, 11]) can effectively facilitate the separation of photogenerated electrons and holes; therefore, PEC water splitting performance of the pristine TiO_2_ can be obviously enhanced. Among various metal oxide semiconductors, hematite (α-Fe_2_O_3_) is regarded as a promising photoanode material because of the suitable band gap (~ 2.0 eV) for sunlight harvesting, excellent stability, and low cost [12]. In addition, the theoretical power conversion efficiency (PCE) of α-Fe_2_O_3_ can reach 15.3%, with a photocurrent density of 12.6 mA cm^− 2^ at 1.23 V vs. reversible hydrogen electrode (RHE) under the standard sun irradiation [13]. Therefore, constructing α-Fe_2_O_3_/TiO_2_ heterostructured photoanode cannot only enhance the carrier separation performance in TiO_2_ but also effectively extend the light absorption range of TiO_2_. Meanwhile, according to some latest research, α-Fe_2_O_3_ photoanodes suffer from short electron-hole pair lifetime and hole diffusion length (2–4 nm), which results in high recombination rate of photogenerated carriers, hindering the improvement of the PEC performance [12]. In that case, to further enhance the PEC water splitting performance of Fe_2_O_3_/TiO_2_ photoanodes, some narrow band gap semiconductors, like CdS [14, 15] and PbS [16], can be coupled to facilitate the separation of photogenerated carriers. Among them, CdS/Fe_2_O_3_/TiO_2_ heterostructured photoanode is considered to be a promising choice with matched band gap and expanded light absorption range. Also, carrier transport process can be effectively improved because photogenerated carriers can be quickly separated at the interface of CdS/Fe_2_O_3_/TiO_2_, thereby greatly decreasing the carrier recombination rates.

What is more, in order to construct an advanced electrode for PEC water splitting system, the electrode materials should possess the characteristics like sufficient incident light capture capability and tunnels for charge transport. Comparing with general planar photoanodes, one dimensional (1D) nanorod (NR) array photoanodes exhibit good incident light harvesting performance due to the enhanced multi-scattering processes [17], which would lead to an enhanced PEC water oxidation performance. Besides, it is reported that 1D NR array also exhibits excellent carrier transport performance since the photogenerated carriers can directly transport along the NR, thus direct carrier recombination at the crystal boundary can be effectively avoided [18]. Also, in order to further enlarge the surface area of such 1D NR arrays, which can bring more PEC reaction sites and enhance the PEC performance, 1D NR with branched nanostructures is expected [19]. Such integrated architecture offers a long optical path for effective light harvesting, short diffusion distance for excellent charge transport, and large surface area for fast interfacial charge collection, which is of great benefit for the enhancement of PEC performance. Hence, it would be of particular interest to design a CdS-modified Fe_2_O_3_/TiO_2_ heterostructure NR array for PEC water oxidation.

Herein, we reported a facile successive ionic layer adsorption and reaction (SILAR)-hydrothermal method to synthesize CdS-modified Fe_2_O_3_/TiO_2_ NR array for efficient PEC water oxidation. UV-vis study confirms the CdS/Fe_2_O_3_/TiO_2_ NR array displays excellent optical response performance with an obvious broadened light absorption range. Improved charge transfer process and declined charge recombination rate can be evidenced by means of PL spectrum and EIS plots. Applied as the photoanode for PEC water oxidation, CdS/Fe_2_O_3_/TiO_2_ NR array exhibits greatly enhanced photocurrent density of 0.62 mA cm^− 2^ (1.23 V vs. RHE) in alkaline electrolyte compared with pristine TiO_2_ (0.32 mA cm^− 2^ at 1.23 V vs. RHE). It is believed that the synthesis route and the application of CdS/Fe_2_O_3_/TiO_2_ NR array presently reported is of great importance and can be applied in other photovoltaic and photoelectronic devices.

## Methods

### Preparation of CdS/Fe_2_O_3_/TiO_2_ NR Heterostructured Photoanode

#### Synthesis of TiO_2_ NR Array

To synthesize TiO_2_ NR array on the FTO glass, the FTO was cut into rectangle and ultrasonically cleaned with deionized water, acetone, and ethanol, successively. Then, the FTO was put into the autoclave containing a mixed solution of deionized water (20 ml), hydrochloric acid (20 ml), and titanium isopropoxide (1.1 ml) and baked at 160 °C for 6 h. After the reaction, the FTO was washed with deionized water and ethanol for several times and then was annealed in air at 450 °C for 0.5 h.

#### Synthesis of Fe_2_O_3_/TiO_2_ NR Array

To grow α-Fe_2_O_3_ on TiO_2_ NR, as obtained TiO_2_ NR array was put into a mixed solution of FeCl_3_ (15 ml, 0.1 M) and NaNO_3_ (15 ml, 0.5 M) and then transferred to the autoclave. Heating at 100 °C for 2 h, the autoclave was cooled to room temperature and the FTO substrate was washed with deionized water and ethanol for several times. Finally, the FTO substrate was annealed in air at 450 °C for 1 h.

#### Synthesis of CdS/Fe_2_O_3_/TiO_2_ NR

The obtained α-Fe_2_O_3_/TiO_2_ NR array was pretreated with an ethanol solution of mercaptopropinioc acid (MPA, 0.3 M) overnight at 50 °C and then washed with ethanol to remove the excess MPA. In order to deposit CdS layer, a facile successive ionic layer adsorption and reaction (SILAR) method is applied. Pretreated NR array was successively immersed into four different solutions for 30 s, including Cd(NO_3_)_2_·4H_2_O (ethanol, 0.1 M), pure ethanol, Na_2_S·9H_2_O (methanol, 0.2 M) and pure methanol, respectively. The SILAR process was repeated for five times and then the substrate was washed with methanol to remove the extra CdS.

#### Materials Characterization

The phase structures were characterized by X-ray powder diffractometer (XRD) in a 2θ range of 20 to 80°. The morphology of the products was studied with field emission scanning electron microscopy (FE-SEM) attached energy-dispersive X-ray spectroscopy (EDS). Transmission electron microscopy (TEM) images were collected via Tecnai 20 U-Twin equipment. The absorption and photoluminescence (PL) spectra were tested with TU-1900 and Hitachi U-4100, respectively.

#### Photoelectrochemical Performance Characterization

The PEC water oxidation performance was characterized with CHI660E electrochemical station with a three-electrode mode. The applied electrolyte was consisted of 1M NaOH. Before testing, the system was bubbled with argon for 30 min to remove the electrolyte dissolved gas. The linear sweep voltammograms (LSV) and chronoamperometric I-*t* curves were recorded under standard sunlight illuminations (100 mW cm^− 2^). Mott-Schottky plots were measured in the dark at an AC frequency of 1.0 kHz.

Hereafter, the electrode potential was converted into the RHE potential with the Nernst equation:

1 $$ {E}_{\mathrm{RHE}}={E}_{\mathrm{Ag}/\mathrm{AgCl}}+0.059\ \mathrm{pH}+{E^o}_{\mathrm{Ag}/\mathrm{AgCl}} $$

where *E*_RHE_ was the converted potential vs. RHE, *E*_Ag/AgCl_ was the measured potential vs. the Ag/AgCl electrode, and *E* ^o^_Ag/AgCl_ = 0.1976 V at 25 °C.

## Result and Discussion

### Structure and Morphology Characterization

**Fig. 1:**
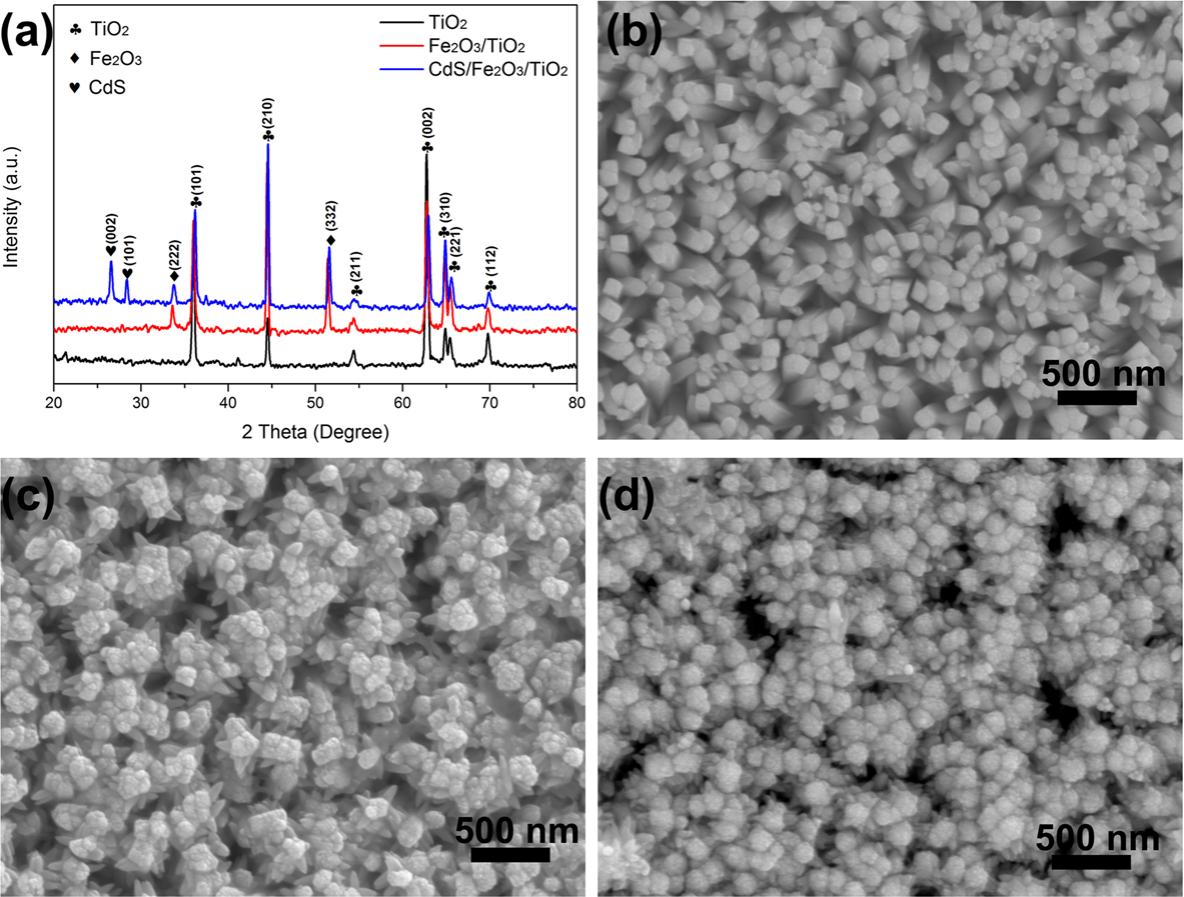
**a** The XRD patterns and **b** SEM images of TiO_2_ NR, Fe_2_O_3_/TiO_2_ NR, and CdS/Fe_2_O_3_

The phase structures of the synthesized products are characterized by the XRD patterns in Fig. 1. As shown in Fig. 1a, the rutile TiO_2_ nanorod arrays (NR) are successfully synthesized. The diffraction peaks at 36.0°, 44.1°, 54.3°, 62.7°, 64.0°, 65.4°, and 69.8° correspond well to (101), (210), (211), (002), (310), (221), and (112) planes of rutile TiO_2_ (JCPDS. 21-1276). After deposition of Fe_2_O_3_, the additional XRD diffraction peaks at 32.9° and 45.2° can be indexed to (222) and (332) planes of Fe_2_O_3_ (JCPDS. 39-0238). SILAR process is applied to grow CdS nanoparticles, the diffraction peaks at 26.4°, 28.2° corresponding well to (002) and (101) planes of CdS (JCPDS. 65-3414) confirm the success growth of CdS nanoparticles on Fe_2_O_3_/TiO_2_. The SEM image in Fig. 1b shows that TiO_2_ NRs are uniformly grown on the FTO substrate with a diameter of 50 nm. The NR surface is relatively smooth. After growth of Fe_2_O_3_ on surface of TiO_2_, the diameter of Fe_2_O_3_/TiO_2_ gests larger and increases to 60 nm. Furthermore, the surface of the NRs gets much rougher. Further deposition of CdS nanoparticles can cause an increase in diameter of the Fe_2_O_3_/TiO_2_ composite NR. To further confirm element distribution of the obtained CdS/Fe_2_O_3_/TiO_2_ NR, the cross-view EDS mapping images are recorded and shown in Additional file 1: Figure S1, Additional file 2: Figure S2. It can be seen that Ti, Fe, Cd, and S elements are uniformly distributed among samples.

**Fig. 2:**
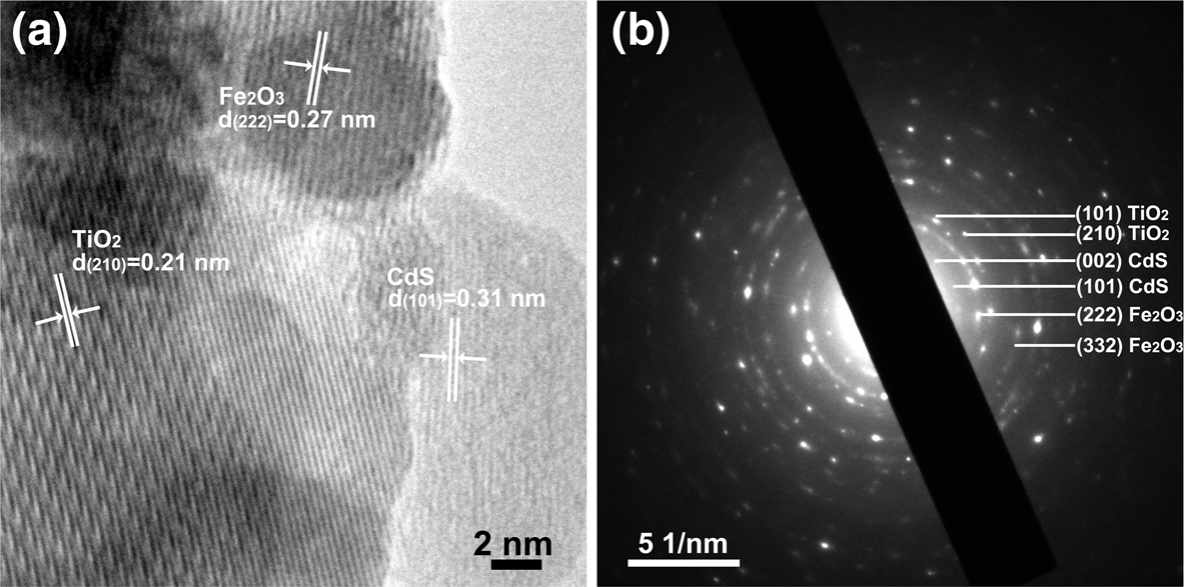
**a** HRTEM image of CdS/Fe_2_O_3_/TiO_2_ NR. The marked d-spacing of 0.31 nm corresponds well to the (101) plane of CdS, the d-spacing of 0.27 nm correspond well to the (222) plane of Fe_2_O_3_ and the d-spacing of 0.21 nm correspond well to the (210) plane of TiO_2_. **b** Selected area electron diffraction pattern of CdS/Fe_2_O_3_/TiO_2_ NR, the diffraction rings correspond to the (002), (101) planes of CdS, (222), (332) planes of Fe_2_O_3_ and (101), (210) planes of TiO_2_

The HRTEM image and selected area electron diffraction (SAED) pattern of CdS/Fe_2_O_3_/TiO_2_ NR are shown in Fig. 2. It can be seen that the both TiO_2_ and Fe_2_O_3_ are well crystallized and the CdS nanoparticles are grown on surface of Fe_2_O_3_. The lattice spacing of 0.31, 0.27, and 0.21 nm can be corresponded well to the (101), (222), and (210) plane of CdS, Fe_2_O_3_, and TiO_2_, respectively (Fig. 2a). The diffraction rings from the recorded SAED pattern in Fig. 2b can be seen, which can be indexed well to (101), (210) planes of rutile TiO_2_, (222), (332) planes of Fe_2_O_3_, and (002), (101) planes of CdS, respectively. The TEM results are in good agreement with the XRD characterization results.

**Fig. 3:**
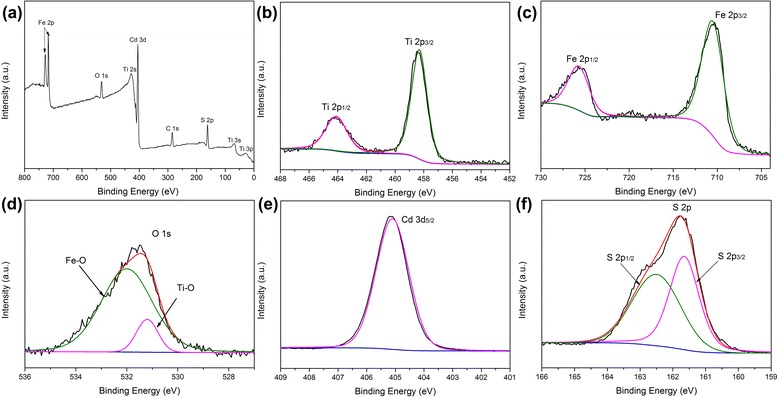
**a** XPS spectra of CdS/Fe_2_O_3_/TiO_2_ NR sample, **b** XPS spectra of Ti 2p including Ti 2p_1/2_ and Ti 2p_3/2_, **c** XPS spectra of Fe 2p including Fe 2p_1/2_ and Fe 2p_3/2_, **d** XPS spectra of O 1s including Fe–O bond and Ti–O bond, **e** XPS spectra of Cd 3d_5/2_, and **f** XPS spectra of S 2p including S 2p_1/2_ and S 2p_3/2_

The chemical composition and valence states of the CdS/Fe_2_O_3_/TiO_2_ hybrid NRs are studied by XPS spectra. Figure 3a shows the survey spectra, the existence of Ti, Fe, O, Cd, and S elements are demonstrated. The appearance of element C is assigned to the carbon-based containment. For the Ti 2p XPS spectrum in Fig. 3b, these splitted two distinct peaks at 458.2 and 464.2 eV can be assigned to Ti 2 p_3/2_ and 2 p_1/2_ of TiO_2_ [20]. The XPS spectrum of Fe 2p is shown in Fig. 3c. Two distinct peaks at 710.6 and 724.10 eV can be seen, which correspond well to Fe 2 p_3/2_ and 2 p_1/2_ peaks of α-Fe_2_O_3_ [21]. The core level XPS spectrum of O 1s is shown in Fig. 3d, where the peak at 531.2 eV is attributed to the Ti–O bond between titanium and oxygen, and the peak at 531.9 eV can be attributed to the Fe–O bond between iron and oxygen [20, 21]. Figure 3e shows XPS spectrum of Cd, which is attributed to the Cd 3d_5/2_ at 405.2 eV. The XPS spectrum of S 2P is shown in Fig. 3f [22]. The center peak is splitted into two peaks of S 2p_1/2_ and 2p_3/2_ at 161.5 and 162.6 eV [22].

**Fig. 4:**
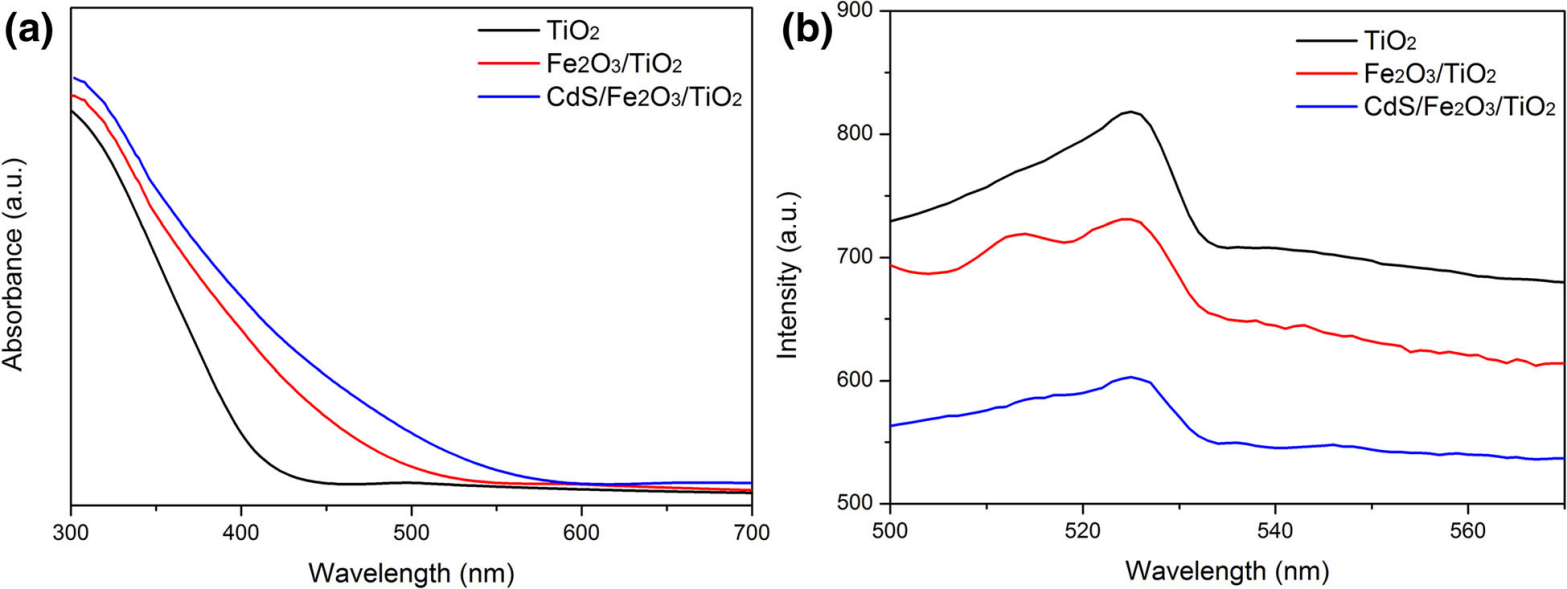
**a** UV-vis absorption spectra and **b** PL spectra of TiO_2_ NR, Fe_2_O_3_/TiO_2_ NR, and CdS/Fe_2_O_3_/TiO_2_ NR samples

Figure 4a shows the absorption spectra of different photoelectrodes. TiO_2_ shows a typical absorption band edge at 400 nm, which can be attributed to the intrinsic band gap absorption of TiO_2_ (3.2 eV). After coupling with Fe_2_O_3_, Fe_2_O_3_/TiO_2_ shows enhanced absorption in the visible light region at about 540 nm. The extension of absorption band edge is due to the visible-sensitive component of Fe_2_O_3_ (2.0–2.2 eV). After further modification of CdS nanoparticles, the light absorption edge can be further extended to 580 nm. It confirms that coupling TiO_2_ with Fe_2_O_3_ and CdS can effectively tune the light absorption property to visible light region. Photoluminescence (PL) spectrum is applied to study the influence of incorporation of CdS and Fe_2_O_3_ in the CdS/Fe_2_O_3_/TiO_2_ hybrid on photogenerated carriers’ transport and recombination behavior. The lower the intensity of PL peak, the higher separation efficiency of photogenerated carrier pairs in the samples. Figure 4b shows the PL spectra of TiO_2_, Fe_2_O_3_/TiO_2_, and CdS/Fe_2_O_3_/TiO_2_ samples. It is obvious that Fe_2_O_3_/TiO_2_ NR achieves lower carrier recombination rate than pristine TiO_2_, and CdS/Fe_2_O_3_/TiO_2_ NR achieves the best carrier transport performance.

In order to further confirm this conclusion, the picosecond-resolved fluorescence transient plots are tested and shown as Additional file 3: Figure S3. The average lifetime *τ* is calculated according to *τ* = (B_1_τ_1_ [2] + *B*_2_ *τ*_2_ [2])/(B_1_τ_1_ + B_2_τ_2_) and the time constant of the fluorescence transients at 511 nm is listed in the Additional file 4: Table S1 [23]. It can be seen that after modifying pristine TiO_2_ with Fe_2_O_3_, the photogenerated carrier lifetime is prolonged. Coupled with CdS, the carrier lifetime can be further enhanced. This result obviously demonstrates the charge separation performance can be effectively enhanced by forming CdS/Fe_2_O_3_/TiO_2_ multi-junction.

**Fig. 5:**
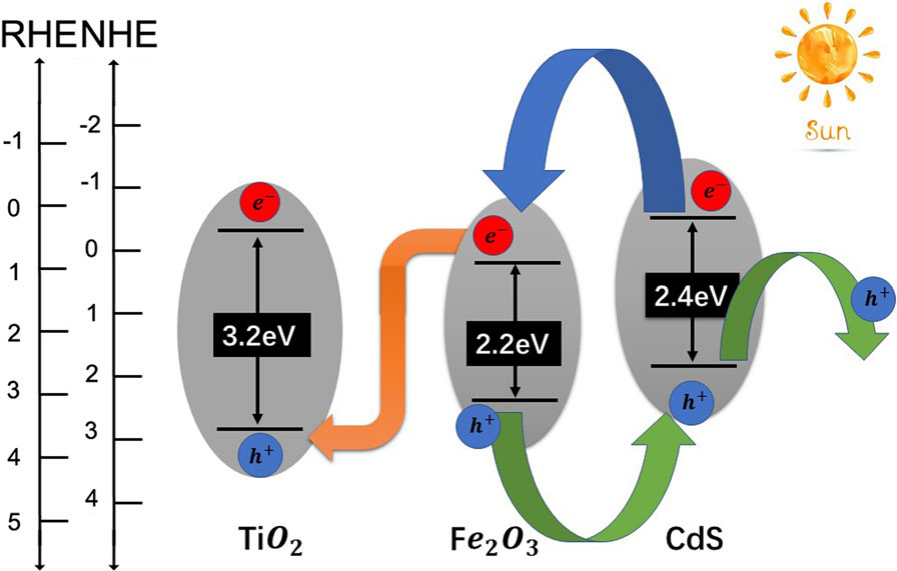
The relative band positions of CdS/Fe_2_O_3_/TiO_2_ ternary system

The possible carrier transport process is illustrated in Fig. 5. In the CdS/Fe_2_O_3_/TiO_2_ ternary system, because both the conduction band position and valence band position of CdS are higher than that of Fe_2_O_3_, the photoinduced electrons in CdS will be transported to conduction band of Fe_2_O_3_, while the photoinduced holes in valence band in Fe_2_O_3_ will be transported to CdS. For the designed abnormal type-II heterostructure between Fe_2_O_3_/TiO_2_, the conduction band position of Fe_2_O_3_ is higher than that of TiO_2_. Under sunlight illumination, photoexcited electron-hole pairs will generate both in TiO_2_ and Fe_2_O_3_. Photogenerated electrons in the conduction band of Fe_2_O_3_ will immediately move to the valence band of TiO_2_ to recombine with the photogenerated holes, thus greatly improving the separation efficiency of photogenerated holes within Fe_2_O_3_ and enhances the photogenerated electron injection efficiency in TiO_2_ [24, 25]. It implies that the coupling of TiO_2_ with Fe_2_O_3_ and CdS can effectively reduce the recombination rate of the photogenerated carrier pairs. Meanwhile, the photogenerated electrons in TiO_2_ move to the counter electrode where the reduction reaction takes place. So, the abnormal type-II heterostructure between Fe_2_O_3_/TiO_2_ plays an important role in the enhanced PEC water oxidation performance.

**Fig. 6:**
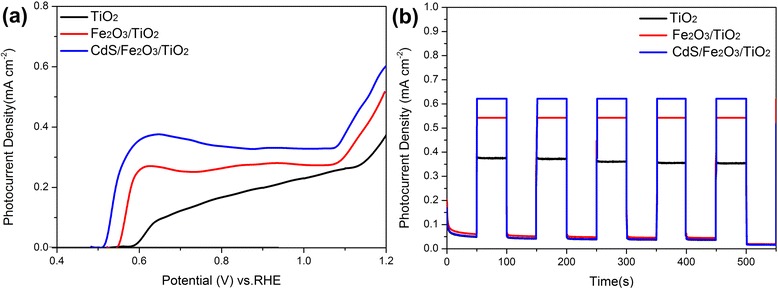
**a** LSV curves of TiO_2_ NR, Fe_2_O_3_/TiO_2_ NR, and CdS/Fe_2_O_3_/TiO_2_ NR samples illumination in 1M NaOH, **b** chronoamperometric I-*t* curves at a bias potential of 1.2 V under chopped illumination

Figure 6 depicts linear sweep voltammograms (LSV) and chronoamperometric I-*t* curves of CdS/Fe_2_O_3_/TiO_2_, Fe_2_O_3_/TiO_2_, and TiO_2_ samples. As shown in Fig. 6a, the photocurrent density of photoanodes under illumination gradually increases after coupling with α-Fe_2_O_3_ and CdS nanoparticles, and the CdS/Fe_2_O_3_/TiO_2_ NR sample exhibits the largest photocurrent density of 0.61 mA cm^− 2^ at 1.2 V vs. RHE, which is almost twice of bare TiO_2_ sample. I-*t* curves at a bias potential of 1.2 V vs. RHE under chopped illumination are shown in Fig. 6b, it can be seen that the samples remain excellent stability and good optical-response property under chopped illumination. CdS/Fe_2_O_3_/TiO_2_ NR sample maintains a photocurrent density of about 0.6 mA cm^− 2^, which is in accordance with the LSV curves.

**Fig. 7:**
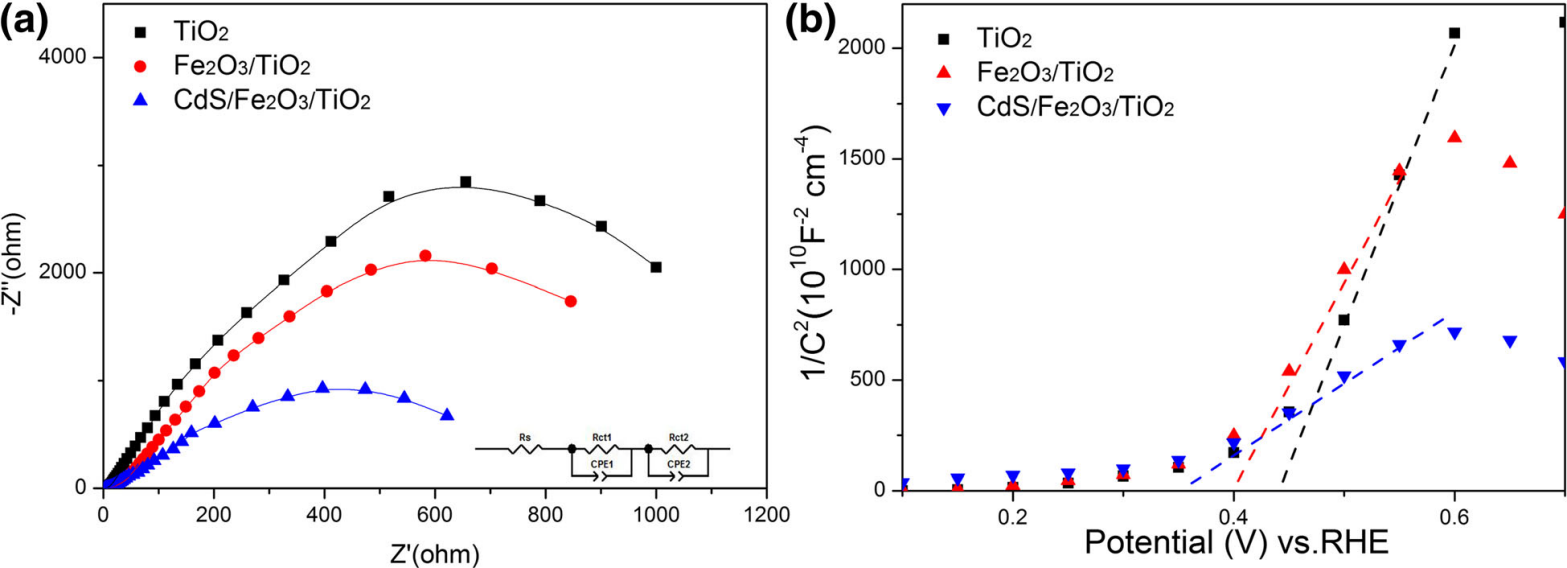
**a** EIS spectra measured at a bias potential of 1.2 V under illumination and **b** Mott-Schottky plots collected at a frequency of 1 KHz in the dark for the TiO_2_ NR, Fe_2_O_3_/TiO_2_ NR, and CdS/Fe_2_O_3_/TiO_2_ NR samples

EIS measurement is performed under illumination and the Nyquist plots are shown in Fig. 7a and Additional file 5: Figure S4. They demonstrate that the Nyquist plots have two semicircles with a contact series resistance (*R*_*s*_) on the FTO substrate. The small semicircle in the Nyquist plots is attributed to the charge transport resistance at the electrode/electrolyte interface, and the large semicircle represents the charge transfer resistance related to the electron transport/recombination within the photoanode materials. The sheet resistance (*R*_*s*_) of the substrate, the charge transfer resistance of the counterelectrode (*R*_ct1_), and the charge transfer resistance (*R*_ct2_) were simulated by the Zview software and the corresponding data are shown in Additional file 6: Table S2. The fitted *R*_*s*_ and *R*_ct1_ values for all samples are similar due to the same configuration and growing substrates are applied, while the *R*_ct2_ values show obviously variation of 1079.5, 880.6, and 679.5 Ω for TiO_2_, Fe_2_O_3_/TiO_2_, and CdS/Fe_2_O_3_/TiO_2_, respectively. It can be seen that after modifying TiO_2_ with Fe_2_O_3_ and CdS, the interfacial charge transfer kinetics are greatly enhanced.

The Mott-Schottky plots of the as obtained samples are listed in Fig. 7b. The slopes determined from the Mott-Schottky plots are used to estimate the carrier density according to the following equation [26]:

Nd = $$ \frac{2}{e_0{\varepsilon \varepsilon}_0}\times \left[\frac{dV}{d\left(1,/,{C}^2\right)}\right] $$

where *e*_0_ is the electronic charge, *ε* is the dielectric constant of the sample, *ε*_0_ is the permittivity of the vacuum, Nd is the donor density, and *V* is the applied voltage. In general, relatively smaller the slope represents higher carrier density.

The flat band potential can be estimated by the following equation:

$$ \frac{1}{C^2}=\frac{2}{e_0{\varepsilon \varepsilon}_0\mathrm{Nd}}\times \left[E-{E}_{\mathrm{fb}}-\frac{kT}{e}\right] $$

The flat band potential (*E*_fb_) is determined by taking the *x* intercept of a linear fit to the Mott-Schottky plot, *1/C* ^*2*^, as a function of applied potential (*E*). Additionally, a remarkable cathodic shift in the flat potential from 0.44 V for TiO_2_ sample to 0.36 V for the CdS/Fe_2_O_3_/TiO_2_ NR sample was observed. This suggests a larger accumulation of electrons in the heterojunction and reflects decreased charge recombination.

It should be noticed that the PEC water oxidation performance of as synthesized CdS/ Fe2O3/TiO2 sample is comparable to some related works. For instance, Sharma et al. reported Fe-TiO_2_/Zn-Fe_2_O_3_ thin films with a performance of 0.262 mA cm^− 2^ at 0.95 V (vs. SCE) [27], while the FTO/Fe_2_O_3_/ZnFe_2_O_4_ photoanode achieves a photocurrent density of 0.4 mA cm^− 2^ [28]. In addition, for the reported Fe_2_O_3_/TiO_2_ nanotube photoanodes, a photocurrent density of 0.5 mA cm^− 2^ is achieved [29, 30]. Comparing with the related works, it can be seen that obtained CdS/Fe_2_O_3_/TiO_2_ photoanode does obtain outstanding and reliable PEC water splitting performance here.

## Conclusions

In conclusion, a facile successive ionic layer adsorption and reaction (SILAR)-hydrothermal method is developed to fabricate CdS-modified Fe_2_O_3_/TiO_2_ NR array for efficient PEC water oxidation. UV-vis study confirms the CdS/Fe_2_O_3_/TiO_2_ NR array displays excellent optical response performance with an obvious broadened light absorption range. Applied as the photoanode for PEC water oxidation, CdS/Fe_2_O_3_/TiO_2_ NR array photoanode exhibits greatly enhanced photocurrent density of 0.62 mA cm^− 2^ (1.23 V vs. RHE) in alkaline electrolyte compared with pristine TiO_2_ (0.32 mA cm^− 2^ at 1.23 V vs. RHE).

## Additional Files

Additional file 1: Figure S1. Cross-sectional SEM image of CdS/Fe_2_O_3_/TiO_2_ NR. (JPEG 742 kb)

Additional file 2: Figure S2. The cross-view EDS mapping images of CdS/Fe_2_O_3_/TiO_2_ NR in Fig. S1. (JPEG 585 kb)

Additional file 3: Figure S3. The picosecond-resolved fluorescence transients of TiO_2_, Fe_2_O_3_/TiO_2_ and CdS/Fe_2_O_3_/TiO_2_ samples. (JPEG 1602 kb)

Additional file 4: Table S1. Dynamics of picosecond-resolved fluorescence transients of TiO_2_, Fe_2_O_3_/TiO_2_ and CdS/Fe_2_O_3_/TiO_2_ samples. (DOCX 11 kb)

Additional file 5: Figure S4. The amplified Nyquist plot of the obtained TiO_2_, Fe_2_O_3_/TiO_2_ and CdS/Fe_2_O_3_/TiO_2_ photoanodes. (JPEG 933 kb)

Additional file 6: Table S2. Series resistance of the obtained TiO_2_, Fe_2_O_3_/TiO_2_ and CdS/Fe_2_O_3_/TiO_2_ photoanodes. (DOCX 13 kb)
