# Supplementary figures and images for: CdS Nanoparticle-Modified α-Fe2O3/TiO2 Nanorod Array Photoanode for Efficient Photoelectrochemical Water Oxidation
Source: Nanoscale Res Lett. 2017 Sep 2;12:520. doi: 10.1186/s11671-017-2278-3 (PMC5581748; doi:10.1186/s11671-017-2278-3)

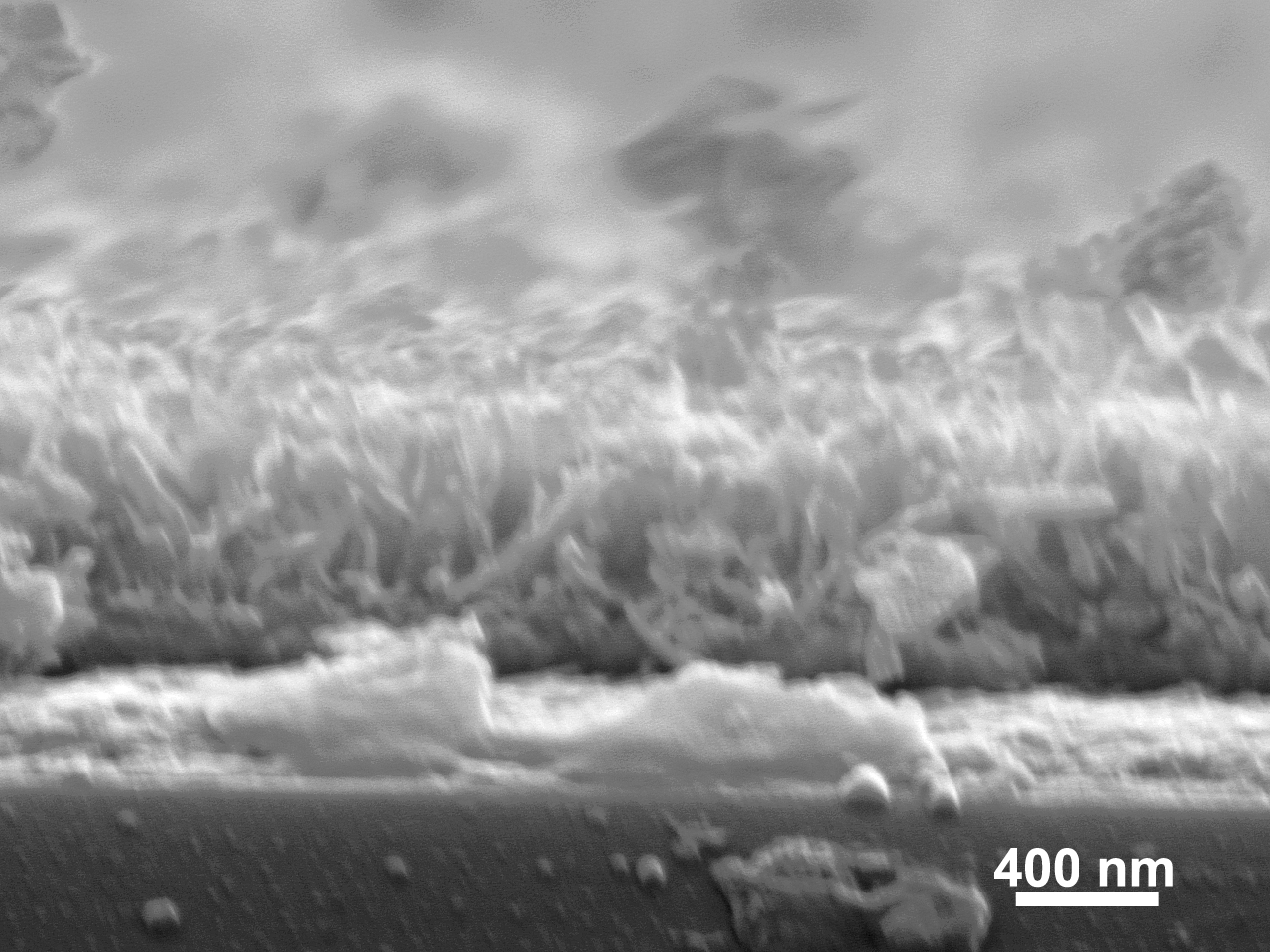

Supplement: Supplementary file 1 — Cross-sectional SEM image of CdS/Fe2O3/TiO2 NR. (JPEG 742 kb) [file 11671_2017_2278_MOESM1_ESM.jpg]

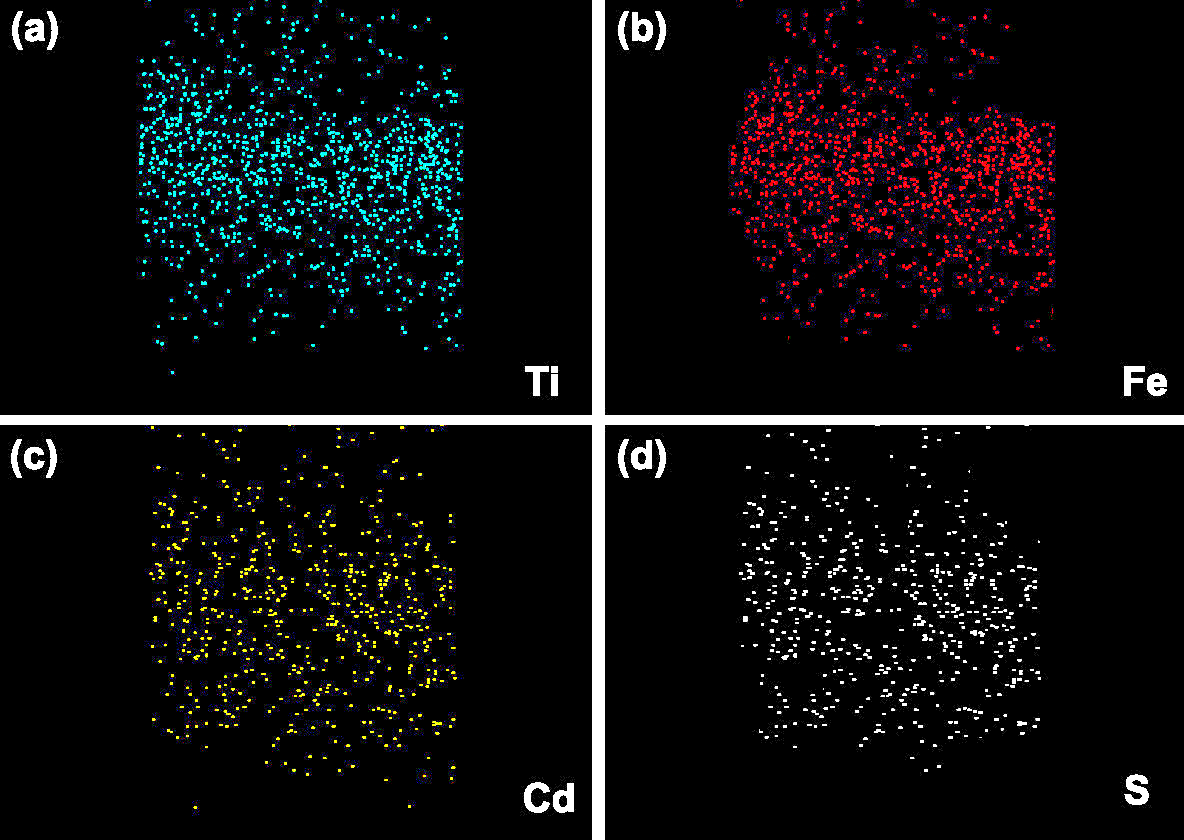

Supplement: Supplementary file 2 — The cross-view EDS mapping images of CdS/Fe2O3/TiO2 NR in Fig. S1. (JPEG 585 kb) [file 11671_2017_2278_MOESM2_ESM.jpg]

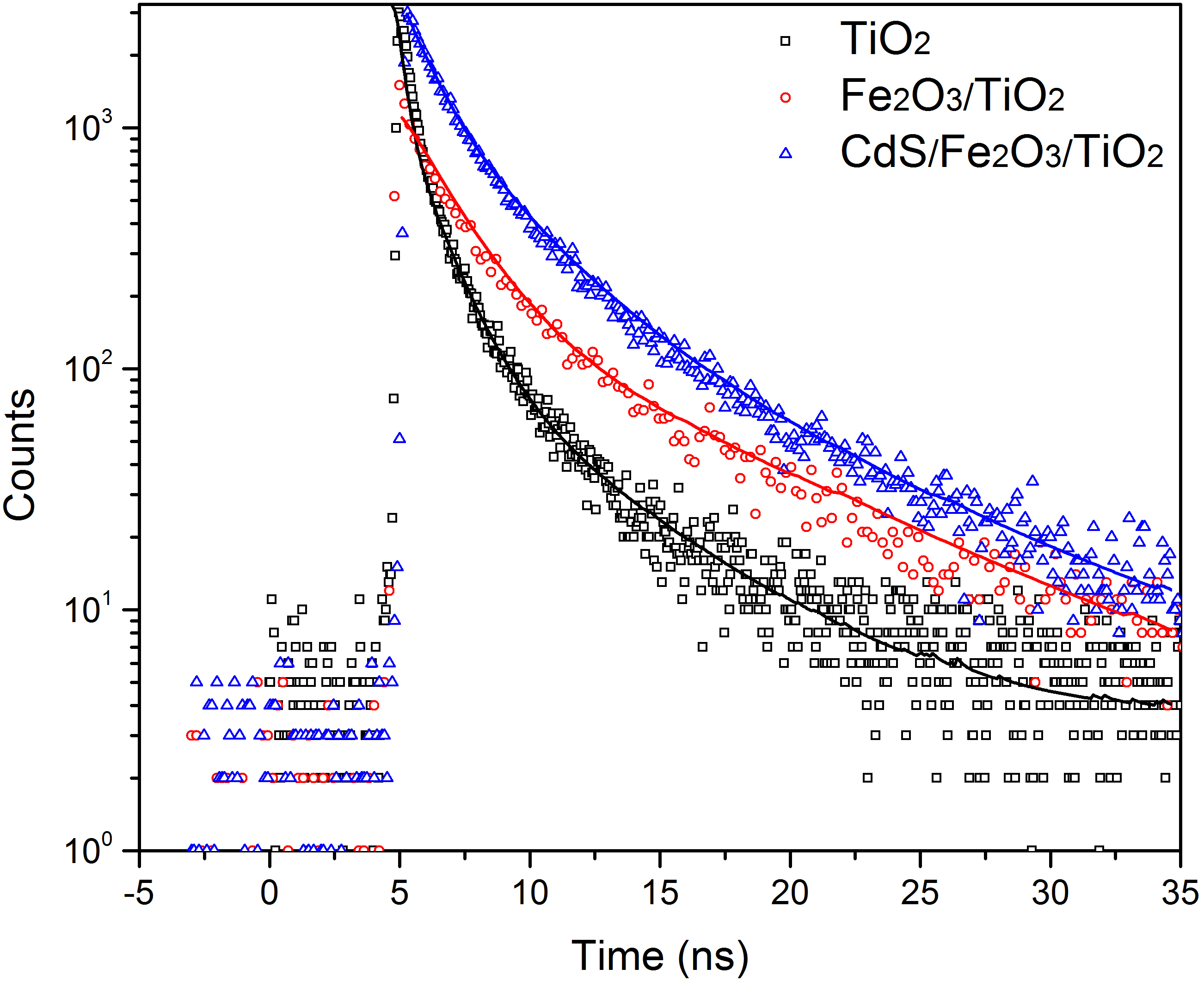

Supplement: Supplementary file 3 — The picosecond-resolved fluorescence transients of TiO2, Fe2O3/TiO2 and CdS/Fe2O3/TiO2 samples. (JPEG 1602 kb) [file 11671_2017_2278_MOESM3_ESM.jpg]

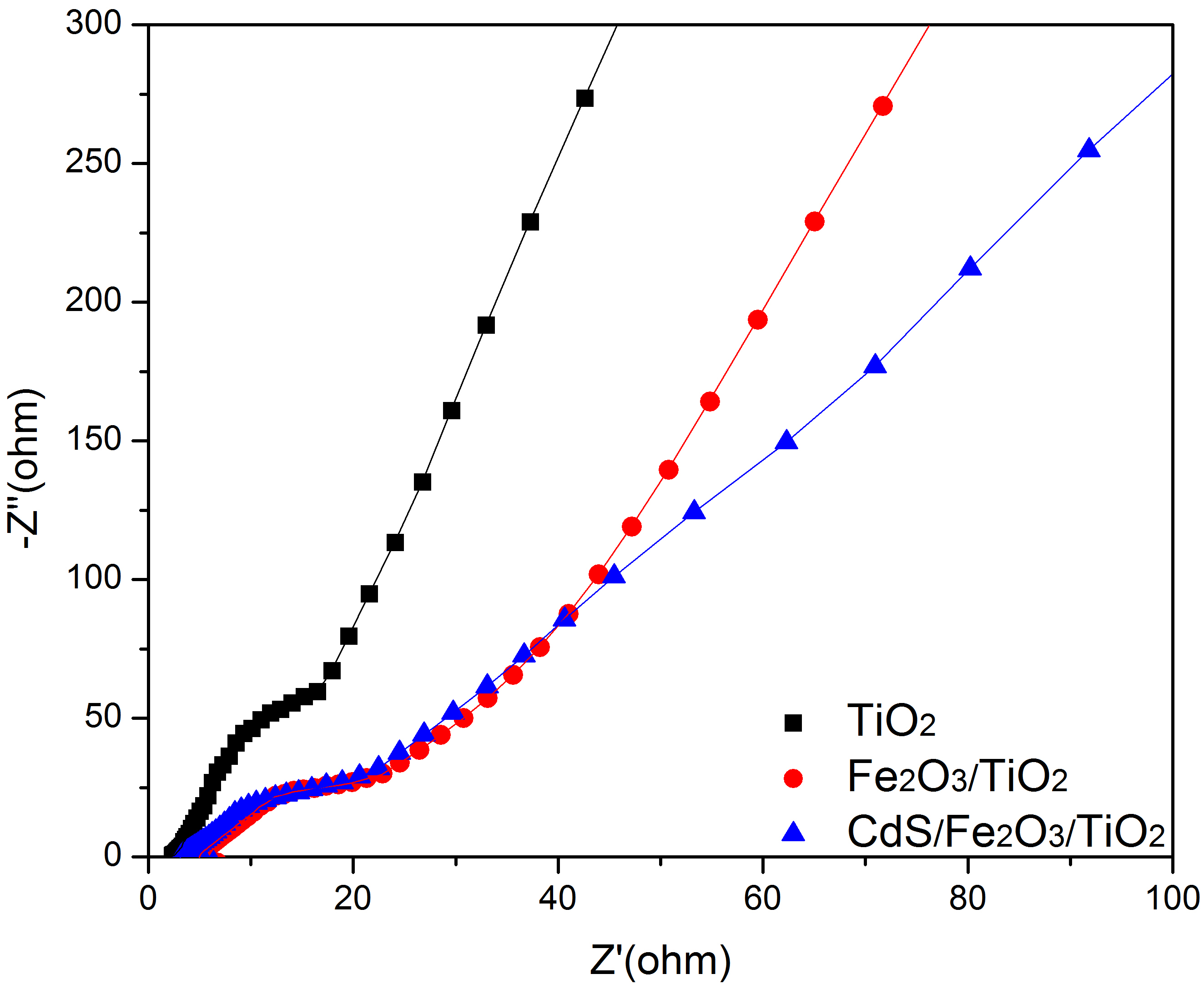

Supplement: Supplementary file 5 — The amplified Nyquist plot of the obtained TiO2, Fe2O3/TiO2 and CdS/Fe2O3/TiO2 photoanodes. (JPEG 933 kb) [file 11671_2017_2278_MOESM5_ESM.jpg]
