# Supplementary material for: CdS Nanoparticle-Modified α-Fe2O3/TiO2 Nanorod Array Photoanode for Efficient Photoelectrochemical Water Oxidation
Source: Nanoscale Res Lett. 2017 Sep 2;12:520. doi: 10.1186/s11671-017-2278-3 (PMC5581748; doi:10.1186/s11671-017-2278-3)
Supplement: Supplementary file 4 — Dynamics of picosecond-resolved fluorescence transients of TiO2, Fe2O3/TiO2 and CdS/Fe2O3/TiO2 samples. (DOCX 11 kb) [file 11671_2017_2278_MOESM4_ESM.docx]

Additional file 5: Table S1 Dynamics of Picosecond-Resolved Fluorescence Transients of TiO_2_, Fe_2_O_3_/TiO_2_ and CdS/Fe_2_O_3_/TiO_2_ samples

| Sample | τ_1_(ns) | τ_2_(ns) | B_1_ | B_2_ | τ(ns) |
| --- | --- | --- | --- | --- | --- |
| TiO_2_ | 1.257 | 3.289 | 44.43 | 54.34 | 2.80 |
| Fe_2_O_3_/TiO_2_ | 1.302 | 5.231 | 65.33 | 34.67 | 3.97 |
| CdS/Fe_2_O_3_/TiO_2_ | 0.4221 | 4.5679 | 47.59 | 52.41 | 4.24 |
