# Supplementary material for: CdS Nanoparticle-Modified α-Fe2O3/TiO2 Nanorod Array Photoanode for Efficient Photoelectrochemical Water Oxidation
Source: Nanoscale Res Lett. 2017 Sep 2;12:520. doi: 10.1186/s11671-017-2278-3 (PMC5581748; doi:10.1186/s11671-017-2278-3)
Supplement: Supplementary file 6 — Series resistance of the obtained TiO2, Fe2O3/TiO2 and CdS/Fe2O3/TiO2 photoanodes. (DOCX 13 kb) [file 11671_2017_2278_MOESM6_ESM.docx]

Additional file 6: Table S2 Series resistance of the obtained TiO_2_, Fe_2_O_3_/TiO_2_ and CdS/Fe_2_O_3_/TiO_2_ photoanodes

| Sample | R_s_ (Ω) | R_ct1_(Ω) | R_ct2_(Ω) |
| --- | --- | --- | --- |
| TiO_2_ | 9.8 | 19.3 | 1079.5 |
| Fe_2_O_3_/TiO_2_ | 10.3 | 21.1 | 880.6 |
| CdS/Fe_2_O_3_/TiO_2_ | 10.1 | 21.5 | 679.5 |
